# Supplementary material for: Variance in Stallion Semen Quality among Equestrian Sporting Disciplines and Competition Levels
Source: Animals (Basel). 2019 Jul 25;9(8):485. doi: 10.3390/ani9080485 (PMC6719077; doi:10.3390/ani9080485)
Supplement: Supplementary file 1 [file animals-09-00485-s001.pdf]

Supplementary File 1

Table S1: Multivariable model fitting

Dependant Variable	Covariates Tested
**MA1:** Above or below industry standards for total volume (ml)	Competing vs. non-competing, discipline, competition level, age, gel-free volume, progressive motility, concentration, total sperm count and total progressively motile sperm
**MA2:** Above or below industry standards for gel-free volume (ml)	Competing vs. non-competing, discipline, competition level, age, total volume, progressive motility, concentration, total sperm count and total progressively motile sperm
**MA3:** Above or below industry standards for progressive motility (%)	Competing vs. non-competing, discipline, competition level, age, total volume, gel-free volume, concentration, total sperm count and total progressively motile sperm
**MA4:** Above or below industry standards for concentration ($x10^6$/ml)	Competing vs. non-competing, discipline, competition level, age, total volume, gel-free volume, progressive motility, total sperm count and total progressively motile sperm
**MA5:** Above or below industry standards for total sperm count ($x10^9$)	Competing vs. non-competing, discipline, competition level, age, total volume, gel-free volume, progressive motility, concentration and total progressively motile sperm
**MA6:** Above or below industry standards for total progressively motile sperm ($x10^9$)	Competing vs. non-competing, discipline, competition level, age, total volume, gel-free volume, progressive motility, concentration and total sperm count

Dependant Variable	Covariates Tested
**MB1:** Above or below industry standards for total volume (ml)	Time between competition and collection, discipline, competition level, age, gel-free volume, progressive motility, concentration, total sperm count and total progressively motile sperm

**MB2:** Above or below industry standards for gel-free volume (ml)	Time between competition and collection, discipline, competition level, age, total volume, progressive motility, concentration, total sperm count and total progressively motile sperm
**MB3:** Above or below industry standards for progressive motility (%)	Time between competition and collection, discipline, competition level, age, total volume, gel-free volume, concentration, total sperm count and total progressively motile sperm
**MB4:** Above or below industry standards for concentration ($x10^6$/ml)	Time between competition and collection, discipline, competition level, age, total volume, gel-free volume, progressive motility and total progressively motile sperm
**MB5:** Above or below industry standards for total sperm count ($x10^9$)	Time between competition and collection, discipline, competition level, age, total volume, gel-free volume, progressive motility, concentration and total progressively motile sperm
**MB6:** Above or below industry standards for total progressively motile sperm ($x10^9$)	Time between competition and collection, discipline, competition level, age, total volume, gel-free volume, progressive motility, concentration and total sperm count

Supplementary File 2

Table S2: Model A1 Results of binary regression analysis, total volume industry standard; B: beta, SE: standard error, P: probability; CI: confidence interval; L: lower; U: upper

**Significant Variables**	**B**	**SE**	**P-values**	**Odds ratio**	**95% Cl (L:U)**
**Discipline**			0.101		
Show Jumping	0.952	0.447	0.033	2.591	1.079:6218
**Semen Parameters**					
Gel-Free Volume	0.221	0.026	$0.01 x10^{-15}$	1.247	1.185: 1.312
Progressive Motility	-0.065	0.020	0.001	0.937	0.901: 0.975
Concentration	-0.016	0.005	0.003	0.984	0.974: 0.995

Total Progressively Motile Sperm	0.511	0.143	0.0003	1.667	1.260: 2.207
**Non-Significant Variables**	**B**	**SE**	**P-values**	**Odds ratio**	**95% Cl (L:H)**
**Competing vs. Non-Competing**			0.498		
Competing	-0.550	0.812	0.498	0.577	0.117: 2.834
Non-Competing	Reference				
**Discipline**			0.101		
Dressage	0.616	0.372	0.098	1.852	0.892: 3.841
Eventing	Reference				
**Competition Level**			0.785		
Lower Level	0.230	0.333	0.490	1.258	0.656: 2.415
Higher Level	0.43	0.382	0.910	1.044	0.494: 2.207
Elite Level	Reference				
**Age Categories**			0.292		
Age 1-4	Reference				
Age 5-9	0.100	0.386	0.795	1.105	0.519: 2.357
Age 10-14	0.420	0.405	0.300	1.522	0.688: 3.369
Age 15-19	0.806	0.793	0.309	2.239	0.474: 10.588
Age 20+	-0.764	0.695	0.272	0.466	0.119: 1.820
**Semen Parameters**					
Total Sperm Count	0.315	0.225	0.163	1.370	0.881: 2.130

Supplementary File 3

Table S3: Model A2 Results of binary regression analysis, gel-free volume industry standard; B: beta, SE: standard error, P: probability; CI: confidence interval; L: lower; U: upper

**Non-Significant Variables**	**B**	**SE**	**P-values**	**Odds ratio**	**95% Cl (L:U)**
**Competing vs. Non-Competing**			**0.992**		
**Competing**	**-17.615**	**1345.303**	**0.990**	**0.000**	**.**
**Non-Competing**	**Reference**				
**Discipline**			**1.000**		
**Show Jumping**	**-4.969**	**471.805**	**0.992**	**0.007**	**.**
**Dressage**	**-1.677**	**474.922**	**0.997**	**0.187**	**.**

Eventing	Reference				
<u>Competition Level</u>			0.999		
Lower Level	-3.290	90.15	0.971	0.037	0.74 x10^{-80} : 1.86 x10^{75}
Higher Level	4.358	509.131	0.993	78.091	.
Elite	Reference				
<u>Age Categories</u>			1.000		
Age 1-4	Reference				
Age 5-9	3.436	328.954	0.992	31.049	0.31 x10^{-280} : 0.31 x10^{282}
Age 10-14	-0.644	228.322	0.998	0.525	0.20 x10^{-247} : 1.41 x10^{245}
Age 15-19	4.957	54302.921	1.000	142.126	.
Age 20+	-0.988	503.454	0.998	0.372	.
<u>Semen Parameters</u>					
Total Sperm Count	515.772	843.528	0.541	0.99 x10^{224}	.
Total Volume	0.349	2.377	0.883	1.418	0.013: 149.52
Progressive Motility	0.402	5.110	0.937	1.495	0.67 x10^{-4} : 3.35 x10^{4}
Concentration	-20.210	33.271	0.544	0.17 x10^{-10}	.
Total Progressively Motile Sperm	-3.489	79.369	0.965	0.031	0.84 x10^{-71}: 1.11 x10^{66}

Table S4: Model A3 Results of binary regression analysis, progressive motility industry standard; B: beta, SE: standard error, P: probability; CI: confidence interval; L: lower; U: upper

Non-Significant Variables	B	SE	P-values	Odds ratio	95% CI (L:U)
<u>Competing vs. Non-Competing</u>			0.811		
Competing	343.765	1100.661	0.755	1.97 x10^{149}	.
Non-Competing	Reference				
<u>Discipline</u>			0.998		
Show Jumping	49.916	1295.818	0.969	4.77 x10^{21}	.
Dressage	-7.256	1792.764	0.997	0.001	.
Eventing	Reference				

<u>**Competition Level**</u>			**0.998**		
**Lower Level**	**-12.527**	**379.558**	**0.974**	**0.4 x10^{-5}**	**.**
**Higher Level**	**-29.771**	**533.098**	**0.955**	**1.18 x10^{-13}**	**.**
**Elite Level**	**Reference**				
<u>**Age Categories**</u>			**1.000**		
**Age 1-4**	**Reference**				
**Age 5-9**	**42.698**	**793.917**	**0.957**	**3.49 x10^{18}**	**.**
**Age 10-14**	**44.600**	**1366.540**	**0.974**	**2.34 x10^{19}**	**.**
**Age 15-19**	**20.729**	**1295.535**	**0.987**	**0.10 x10^{10}**	**.**
**Age 20+**	**-49.317**	**4342.448**	**0.991**	**3.82 x10^{-22}**	**.**
<u>**Semen Parameters**</u>					
**Total Sperm Count**	**-109.576**	**321.825**	**0.733**	**2.58 x10^{-48}**	**.**
**Total Volume**	**0.124**	**8.944**	**0.989**	**1.132**	**2.76 x10^{-8}: 4.65 x10^{7}**
**Gel-Free Volume**	**-0.204**	**8.675**	**0.981**	**0.816**	**3.37 x10^{-8}: 1.98 x10^{7}**
**Concentration**	**0.147**	**0.929**	**0.874**	**1.158**	**0.187: 7.157**
**Total Progressively Motile Sperm**	**307.602**	**831.788**	**0.712**	**3.89 x10^{133}**	**.**

Table S5: Model A4 Results of binary regression analysis, semen concentration industry standard; B: beta, SE: standard error, P: probability; CI: confidence interval; L: lower; U: upper

**Non-Significant Variables**	**B**	**SE**	**P-values**	**Odds ratio**	**95% CI (L:U)**
<u>**Competing vs. Non-Competing**</u>			**0.966**		
**Competing**	**-6.144**	**142.348**	**0.966**	**0.002**	**1.46 x10^{-124}: 3.15 x10^{118}**
**Non-Competing**	**Reference**				
<u>**Discipline**</u>			**0.999**		
**Show Jumping**	**-38.208**	**956.663**	**0.968**	**2.55 x10^{-17}**	**.**
**Dressage**	**-5.534**	**783.746**	**0.994**	**0.004**	**.**
**Eventing**	**Reference**				
<u>**Competition Level**</u>			**1.000**		
**Lower Level**	**-0.427**	**240.903**	**0.999**	**0.652**	**5.72 x10^{-206}: 7.44**

					$x10^{204}$
**Higher Level**	**-4.610**	**354.566**	**0.990**	**0.010**	**1.55 $x10^{-304}$: 6.38 $x10^{299}$**
**Elite Level**	**Reference**				
**<u>Age Categories</u>**			**1.000**		
**Age 1-4**	**Reference**				
**Age 5-9**	**12.195**	**478.135**	**0.980**	**1.98 $x10^{5}$**	**.**
**Age 10-14**	**23.457**	**540.049**	**0.965**	**1.54 $x10^{10}$**	**.**
**Age 15-19**	**11.145**	**1078.064**	**0.992**	**6.92 $x10^{4}$**	**.**
**Age 20+**	**13.150**	**469.149**	**0.978**	**5.14 $x10^{5}$**	**.**
**<u>Semen Parameters</u>**					
**Total Sperm Count**	**88.752**	**290.257**	**0.760**	**3.50 $x10^{38}$**	**3.00 $x10^{-209}$: 4.09 $x10^{285}$**
**Total Volume**	**1.367**	**5.321**	**0.797**	**3.92**	**0.12 $x10^{3}$: 1.33 $x10^{5}$**
**Gel-Free Volume**	**- 39.496**	**129.204**	**0.760**	**7.03 $x10^{-18}$**	**7.39 $x10^{-128}$: 6.69 $x10^{92}$**
**Progressive Motility**	**-0.634**	**10.283**	**0.951**	**0.53**	**9.37 $x10^{-10}$: 3.00 $x10^{8}$**
**Total Progressively Motile Sperm**	**4.872**	**206.873**	**0.981**	**130.64**	**1.06 $x10^{-174}$: 1.61 $x10^{178}$**

Table S6: Model A5 Results of binary regression analysis, total sperm count industry standard; B: beta, SE: standard error, P: probability; CI: confidence interval; L: lower; U: upper

Non-Significant Variables	B	SE	P-values	Odds ratio	95% CI (L:U)
**<u>Competing vs. Non-Competing</u>**			**0.974**		
**Competing**	**72.544**	**2215.907**	**0.974**	**3.20 $x10^{31}$**	**.**
**Non-Competing**	**Reference**				
**<u>Discipline</u>**			**0.922**		
**Show Jumping**	**-5.962**	**93.714**	**0.949**	**0.003**	**4.38 $x10^{-83}$: 1.51 $x10^{77}$**
**Dressage**	**4.769**	**55.384**	**0.931**	**117.819**	**8.48 $x10^{-46}$: 1.64 $x10^{49}$**
**Eventing**	**Reference**				
**<u>Competition Level</u>**			**0.997**		

**Lower Level**	**2.687**	**36.066**	**0.941**	**14.686**	**2.93 x10^{-30}: 7.36 x10^{31}**
**Higher Level**	**-3.760**	**303.718**	**0.990**	**0.023**	**6.94 x10^{-261}: 7.81 x10^{256}**
**Elite Level**	**Reference**				
**<u>Age Categories</u>**			**1.000**		
**Age 1-4**	**Reference**				
**Age 5-9**	**3.370**	**1.04 x10^{4}**	**1.000**	**29.087**	**.**
**Age 10-14**	**8.536**	**1.04 x10^{4}**	**0.999**	**5.10 x10^{3}**	**.**
**Age 15-19**	**56.214**	**1.04 x10^{4}**	**0.996**	**2.59 x10^{24}**	**.**
**Age 20+**	**-121.652**	**5.77 x10^{5}**	**1.000**	**1.47 x10^{-53}**	**.**
**<u>Semen Parameters</u>**					
**Concentration**	**0.003**	**0.061**	**0.956**	**1.003**	**0.891: 1.131**
**Total Volume**	**0.519**	**10555**	**0.739**	**1.680**	**0.080: 35.411**
**Gel-Free Volume**	**0.617**	**3.442**	**0.858**	**1.853**	**0.002: 1.58 x10^{3}**
**Progressive Motility**	**-50.468**	**56.674**	**0.373**	**1.21 x10^{-22}**	**6.94 x10^{-71}: 2.10 x10^{26}**
**Total Progressively Motile Sperm**	**852.517**	**930.644**	**0.360**	**.**	**.**

Supplementary File 4

Table S7: Model A6 Results of binary regression analysis, total progressively motile sperm industry standard; B: beta, SE: standard error, P: probability; CI: confidence interval; L: lower; U: upper

**Significant Variables**	**B**	**SE**	**P-values**	**Odds ratio**	**95% Cl (L:U)**
**<u>Semen Parameters</u>**					
Gel-Free Volume	0.023	0.011	0.038	1.024	1.001: 1.046
Progressive Motility	0.225	0.028	0.009 x10^{-13}	1.252	1.185: 1.323
Total Sperm Count	1.940	0.199	0.01 x10^{-20}	6.960	4.714: 10.275
**Non-Significant Variables**	**B**	**SE**	**P-values**	**Odds ratio**	**95% Cl (L:H)**
**<u>Competing vs. Non-Competing</u>**			0.547		
Competing	0.890	1.478	0.547	2.435	0.134: 44.150
Non-Competing	Reference				
**<u>Discipline</u>**			0.137		
Show Jumping	-0.632	0.630	0.316	0.531	0.155: 1.828

Dressage	0.309	0.623	0.620	1.363	0.402: 4.624
Eventing	Reference				
**Competition Level**			0.905		
Lower Level	0.188	0.449	0.676	1.206	0.500: 2.911
Higher Level	0.183	0.643	0.776	1.200	0.340: 4.233
Elite Level	Reference				
**Age Categories**			0.683		
Age 1-4	Reference				
Age 5-9	-0.399	0.551	0.539	0.731	0.242: 2.098
Age 10-14	-0.138	0.586	0.814	0.871	0.276: 2.748
Age 15-19	-1.633	1.169	0.162	0.195	0.020: 1.931
Age 20+	-1.228	2.685	0.647	0.293	0.002: 56.478
**Semen Parameters**					
Concentration	-0.003	0.003	0.356	0.997	0.990: 1.004
Total Volume	-0.002	0.020	0.900	0.998	0.960: 1.037

Supplementary File 5

Table S8: Model B1 Results of binary regression analysis, total volume industry standard; B: beta, SE: standard error, P: probability; CI: confidence interval; L: lower; U: upper

**Significant Variables**	**B**	**SE**	**P-values**	**Odds ratio**	**95% CI (L:U)**
**Semen Parameters**					
Concentration	-0.066	0.007	0.01 $x10^{-18}$	0.936	0.923: 0.949
Total Sperm Count	1.363	0.145	0.006 $x10^{-18}$	3.908	2.940: 5.194
**Non-Significant Variables**	**B**	**SE**	**P-values**	**Odds ratio**	**95% CI (L:H)**
**Timing Between Competition and Collection**			0.298		
Timing	-0.006	0.005	0.232	0.994	0.984: 1.004
**Discipline**			0.246		
Show Jumping	0.729	0.435	0.094	2.073	0.883: 4.866
Dressage	0.512	0.572	0.371	1.669	0.544: 5.124
Eventing	Reference				
**Competition Level**			0.594		
Lower Level	-0.015	0.619	0.981	0.985	0.293: 3.317
Higher Level	-0.644	0.635	0.311	0.525	0.151: 1.825
Elite Level	Reference				

**Age Categories**			0.310		
Age 1-4	Reference				
Age 5-9	0.767	0.710	0.280	2.154	0.536: 8.663
Age 10-14	1.100	0.742	0.138	3.004	0.702: 12.857
**Semen Parameters**					
Gel-Free Volume	-0.024	0.048	0.623	0.977	0.888: 1.073
Progressive Motility	-0.054	0.035	0.127	0.948	0.884: 1.015
Progressively Motile Sperm	0.082	0.206	0.689	1.086	0.725: 1.625

Supplementary File 6

Table S9: Model B2 Results of binary regression analysis, gel-free volume industry standard; B: beta, SE: standard error, P: probability; CI: confidence interval; L: lower; U: upper

Non-Significant Variables	B	SE	P-values	Odds ratio	95% CI (L:H)
**Timing Between Competition and Collection**			0.996		
Timing	0.029	5.190	0.996	1.030	0.39×10^{4} : 2.69×10^{4}
**Discipline**			1.000		
Show Jumping	16.480	1539.634	0.991	1.44×10^{6}	.
Dressage	-15.442	777.417	0.984	1.97×10^{-4}	.
Eventing	Reference				
**Competition Level**			1.000		
Lower Level	17.139	3852.996	0.996	2.78×10^{7}	.
Higher Level	12.818	1026.842	0.990	3.69×10^{5}	.
Elite Level	Reference				
**Age Categories**			1.000		
Age 1-4	Reference				
Age 5-9	10.796	2289.653	0.996	4.88×10^{4}	.
Age 10-14	13.326	2400.473	0.996	6.13×10^{5}	.
**Semen Parameters**					
Total Volume	0.024	49.373	1.000	1.025	9.64×10^{-43} : 1.09×10^{42}
Progressive Motility	0.097	40.489	0.998	1.102	3.78×10^{-35} : 3.21×10^{34}

Concentration	-4.986	41.366	0.904	0.007	4.20 $x10^{-38}$: 1.11 $x10^{33}$
Total Sperm Count	124.806	1034.679	0.904	1.59 $x10^{54}$	.
Progressively Motile Sperm	-0.854	193.690	0.996	0.426	5.75 $x10^{-166}$: 3.15 $x10^{164}$

Table S10: Model B3 Results of binary regression analysis, progressive motility industry standard; B: beta, SE: standard error, P: probability; CI: confidence interval; L: lower; U: upper

**Non-Significant Variables**	**B**	**SE**	**P-values**	**Odds ratio**	**95% Cl (L:H)**
**Timing Between Competition and Collection**			**0.986**		
**Timing**	**-0084**	**4.752**	**0.986**	**0.920**	**0.000083: 10196.007861**
**Discipline**			**1.000**		
**Show Jumping**	**-8.172**	**1179.451**	**0.994**	**0.000282**	**0.0E0: .**
**Dressage**	**-10.341**	**1237.371**	**0.993**	**0.000032**	**0.0E0: .**
**Eventing**	**Reference**				
**Competition Level**			**1.000**		
**Lower Level**	**9.758**	**4197.490**	**0.998**	**17300.388**	**0.0E0: .**
**Higher Level**	**-8.618**	**2057.511**	**0.997**	**0.000181**	**0.0E0:**
**Elite Level**	**Reference**				
**Age Categories**			**1.000**		
**Age 1-4**	**Reference**				
**Age 5-9**	**9.940**	**3150.416**	**0.997**	**20749.593**	**0.0E0: .**
**Age 10-14**	**15.991**	**2469.675**	**0.995**	**8806933.000**	**0.0E0: .**
**Semen Parameters**					
**Total Volume**	**-0.317**	**24.660**	**0.990**	**0.729**	**7.4464E-22: 7.1282E20**
**Gel-Free Volume**	**0.240**	**49.325**	**0.996**	**1.272**	**1.3154E-42: 1.2295E42**
**Concentration**	**0.096**	**20.312**	**0.996**	**1.101**	**5.6514E-18: 2.1432E17**
**Total Sperm Count**	**-51.219**	**706.532**	**0.942**	**5.7025E-23**	**00.0E0: .**

**Progressively Motile Sperm**	**133.423**	**1663.278**	**0.936**	**8.8119E57**	**0.0E0: .**

Table S11: Model B4 Results of binary regression analysis, semen concentration industry standard; B: beta, SE: standard error, P: probability; CI: confidence interval; L: lower; U: upper

**Non-Significant Variables**	**B**	**SE**	**P-values**	**Odds ratio**	**95% CI (L:H)**
**Timing Between Competition and Collection**			**0.938**		
**Timing**	**-0.0501**	**6.478**	**0.938**	**0.606**	**0.02 $x10^{-4}$: 19.81 $x10^{4}$**
**Discipline**			**0.998**		
**Show Jumping**	**-25.028**	**450.918**	**0.956**	**1.35 $x10^{-11}$**	**.**
**Dressage**	**16.626**	**3785.032**	**0.996**	**1.66 $x10^{7}$**	**.**
**Eventing**	**Reference**				
**Competition Level**			**1.000**		
**Lower Level**	**7.441**	**802.375**	**0.993**	**1704.525**	**.**
**Higher Level**	**-5.120**	**889.757**	**0.995**	**0.006**	**.**
**Elite Level**	**Reference**				
**Age Categories**			**0.997**		
**Age 1-4**	**Reference**				
**Age 5-9**	**-12.382**	**187.324**	**0.947**	**0.4 $x10^{-5}$**	**1.49 $x10^{-165}$: 1.18 $x10^{154}$**
**Age 10-14**	**9.931**	**566.338**	**0.986**	**2.06 $x10^{4}$**	**.**
**Semen Parameters**					
**Total Volume**	**3.225**	**133.184**	**0.981**	**25.164**	**1.08 $x10^{-112}$: 5.86 $x10^{114}$**
**Gel-Free Volume**	**- 131.445**	**384.519**	**0.732**	**8.21 $x10^{-58}$**	**.**
**Progressive Motility**	**-0.009**	**3.539**	**0.998**	**0.991**	**0.001: 10.19 $x10^{2}$**
**Total Sperm Count**	**525.986**	**1538.495**	**0.732**	**2.71 $x10^{228}$**	**.**
**Total Progressively Motile Sperm**	**-0.180**	**160.892**	**0.999**	**0.836**	**9.34 $x10^{-138}$: 7.47 $x10^{136}$**

Table S12: Model B5 Results of binary regression analysis, total sperm count industry standard; B: beta, SE: standard error, P: probability; CI: confidence interval; L: lower; U: upper

**Non-Significant Variables**	**B**	**SE**	**P-values**	**Odds ratio**	**95% CI (L:H)**
**Timing Between Competition and Collection**			**0.929**		
**Timing**	**-0.291**	**3.264**	**0.929**	**0.748**	**0.001: 448.835**
**Discipline**			**1.000**		
**Show Jumping**	**61.664**	**4972.086**	**0.990**	**6.03 x10^{26}**	**.**
**Dressage**	**34.612**	**4376.208**	**0.994**	**1.07 x10^{15}**	**.**
**Eventing**	**Reference**				
**Competition Level**			**0.999**		
**Lower Level**	**19.910**	**446.731**	**0.964**	**4.44 x10^{8}**	**.**
**Higher Level**	**-0.085**	**3824.128**	**1.000**	**0.918**	**.**
**Elite Level**	**Reference**				
**Age Categories**			**1.000**		
**Age 1-4**	**Reference**				
**Age 5-9**	**0.523**	**896.402**	**1.000**	**1.687**	**.**
**Age 10-14**	**4.997**	**1480.570**	**0.997**	**147.897**	**.**
**Semen Parameters**					
**Total Volume**	**0.821**	**17.740**	**0.963**	**2.272**	**1.80 x10^{-15} : 2.86 x10^{15}**

Supplementary File 7
Table S13: Model B6 Results of binary regression analysis, total progressively motile sperm industry standard; B: beta, SE: standard error, P: probability; CI: confidence interval; L: lower; U: upper

**Significant Variables**	**B**	**SE**	**P-values**	**Odds ratio**	**95% CI (L:U)**
**Semen Parameters**					
Progressive Motility	0.204	0.049	0.029 x10^{-3}	1.226	1.114: 1.349
Total Sperm Count	0.097	0.405	0.014 x10^{-10}	9.947	4.499: 21.990
**Non-Significant Variables**	**B**	**SE**	**P-values**	**Odds ratio**	**95% CI (L:H)**
**Time Between Competition and Collection**			0.610		
Timing	-0.005	0.010	0.610	0.995	0.976: 1.014
**Discipline**			0.293		
Show Jumping	0.863	0.880	0.327	2.369	0.422: 13.293
Dressage	-1.148	1.378	0.405	0.317	0.021: 4.730

Eventing	Reference				
**Competition Level**			0.859		
Lower Level	-0.319	1.217	0.793	0.727	0.067: 7.893
Higher Level	0.306	1.158	0.791	1.359	0.141: 13.133
Elite Level	Reference				
**Age Categories**			0.516		
Age 1-4	Reference				
Age 5-9	1.298	1.290	0.314	3.006	0.293: 45.876
Age 10-14	1.547	1.356	0.254	4.699	0.330: 67.004
**Semen Parameters**					
Total Volume	-0.004	0.028	0.898	0.996	0.944: 1.052
Gel-Free Volume	0.038	0.028	0.168	1.039	0.984: 1.097
Concentration	0.000327	0.007	0.964	1.000	0.986: 1.015
